# Evaluating a Sexual Health Patient Education Resource

**DOI:** 10.6004/jadpro.2015.6.3.6

**Published:** 2015-05-01

**Authors:** Marianne Matzo, Sandi Troup, Kamal Hijjazi, Betty Ferrell

**Affiliations:** 1 College of Nursing and Stephenson Cancer Center, University of Oklahoma Sciences Center, Oklahoma City, Oklahoma;; 2 Stephenson Cancer Center, University of Oklahoma Sciences Center, Oklahoma City, Oklahoma;; 3 College of Nursing, University of Oklahoma Health Sciences Center, Oklahoma City, Oklahoma;; 4 City of Hope, Los Angeles, California

## Abstract

This article shares the findings of an evaluation of a patient teaching resource for sexual health entitled *Everything Nobody Tells You About Cancer Treatment and Your Sex Life: From A to Z*, which was accomplished through systematic conceptualization, construction, and evaluation with women diagnosed with breast or gynecologic cancer. This resource, which has evolved from patient-focused research and has been tested in the clinical setting, can be used in patient education and support. Oncology professionals are committed to addressing quality-of-life concerns for patients across the trajectory of illness. Sexuality is a key concern for patients and impacts relationships and overall quality of life. Through careful assessment, patient education, and support, clinicians can ensure that sexuality is respected as an essential part of patient-centered care.

Findings from research studies regarding cancer survivorship indicate that compromised sexuality and intimacy, common in cancer care, can lead to diminished quality of life (QOL; Matzo & Hijjazi, 2009). Approximately 50% of women who have had long-term treatment for breast and reproductive organ cancers report long-term sexual problems (Matzo, Ehiemua Pope, & Whalen, 2013). There are insufficient data regarding what information patients want from their oncology care providers regarding their sexual health, when they want help, to what degree they value such information, and how they would find help to be of most use. There are also few empiric data that inform oncology professionals of this information, how to assess it, and how to care for patients regarding their sexual health (Burbie & Polinsky, 1992; Cummins, Deeks, & McCabe, 2000; McMillan & Mahon, 1994; Miller, Montie, & Wei, 2005; O’Boyle & Waldron, 1997; Robinson & Molzahn, 2007; Rubenstein et al., 1995; Rummans et al., 2006).

Our previously published article reported on a sexual health educational guide for women diagnosed with ovarian cancer, developed as part of a grant from the American Cancer Society (Matzo, Graham, Troup, & Ferrell, 2014). Women in the pilot study were asked what they thought would have been helpful to them regarding maintaining sexual health as they went through cancer treatment. They identified two primary areas: education and communication.

Regarding education, they wished that they had been better informed about what to expect regarding possible sexual health alterations to be more proactive in sexual health maintenance following their cancer treatments. They indicated that the educational materials available to them in their oncologists’ offices did not meet their educational needs and that the information that was available was given to them too late, generally after treatment was completed and many sexual problems had been experienced.

In addition, women in the pilot study voiced the need for better communication with their professional caregivers regarding sexual health needs or concerns to prevent complications (e.g., damaged and dry vaginal tissue and subsequent pain). They also expressed their struggle to communicate new intimate sexual challenges to their health-care practitioners (HCPs) and their partners.

An Institute of Medicine Report (IOM, 2007) addressed cancer care for the whole patient. It stated that to ensure appropriate psychosocial health, HCPs should facilitate effective communication between patients and care providers. In reality, this comprehensive care including sexual health often does not occur. One study of an oncology population documented that 28% of the patients indicated that their physicians did not pay attention to anything other than their medical needs (Young, 2007). Another study documented that for women with ovarian cancer, their satisfaction with care for sexual problems was lower than for their overall cancer care ( Lindau et al., 2007).

Many cancer survivors report that they were not prepared for the changes in their sex lives (Matzo, Ehiemua Pope, & Whalen, 2013). Cancer and its treatment can lead to sexual and reproductive dysfunction, loss of self-esteem, depression, and disruption of crucial supportive relationships (Krychman, Pereira, Carter, & Amsterdam, 2006; Robinson & Molzahn, 2007., 2014). It has also been documented that anticipation and validation of patients’ concerns serve to alleviate emotional suffering (Burbie & Polinsky, 1992; Cummins et al., 2000; McMillan & Mahon, 1994; Miller et al., 2005; O’Boyle & Waldron, 1997; Robinson & Molzahn, 2007; Rubenstein et al., 1995; Rummans et al., 2006). An extensive literature review and our study findings documented that patients often felt that sexuality had been ignored in their cancer care (Matzo et al., 2013). In response to our study participants’ needs, we developed the sexual health education resource *Everything Nobody Tells You About Cancer Treatment and Your Sex Life: From A to Z* (hereafter referred to as *The A to Z Guide*; Matzo et al., 2014).

*The A to Z Guide* was designed to address an unmet need regarding the availability of a short, comprehensive, user-friendly guide that offered women specific information regarding maintaining sexual health after their cancer diagnosis. This article reports the findings of an evaluation study of this guide. We conducted a single-point design to determine whether an educational tool developed from the findings of individual interviews and focus groups had achieved the intended outcomes and to make revisions based on these findings. An electronic version of *The A to Z Guide* can be downloaded and printed for patient use from http://kanwa.org/sexual-health/a-z-guide/.

## METHODS

The first draft of *The A to Z Guide* was distributed to the women who participated in our focus groups and in individual interviews, and they were asked to review and edit it. Those changes were incorporated, and the second iteration of the guide was given to a local ovarian cancer support group for feedback and edits. This article reports on the process of evaluating the third iteration utilizing an online assessment.

Eligible reviewers (women diagnosed with either breast or gynecologic cancer) were provided with a link to our study materials (*The A to Z Guide* and electronic questionnaire) accessed through our collaborators’ websites. Women were requested to read the online consent for participation, after which they could access *The A to Z Guide* and complete the questionnaire evaluation online. The evaluation tool (Figure) separated out educational domains into five categories: impacts of treatments, surgeries, and cancer itself; communication; intimacy; self-care (wellness); and self-care (vaginal health). Each of these domains included a question regarding the degree to which the woman understood the material presented. Women were asked to rate each question on a scale of 1 to 5, with 1 being poor and 5 being excellent.

**Figure 1:**
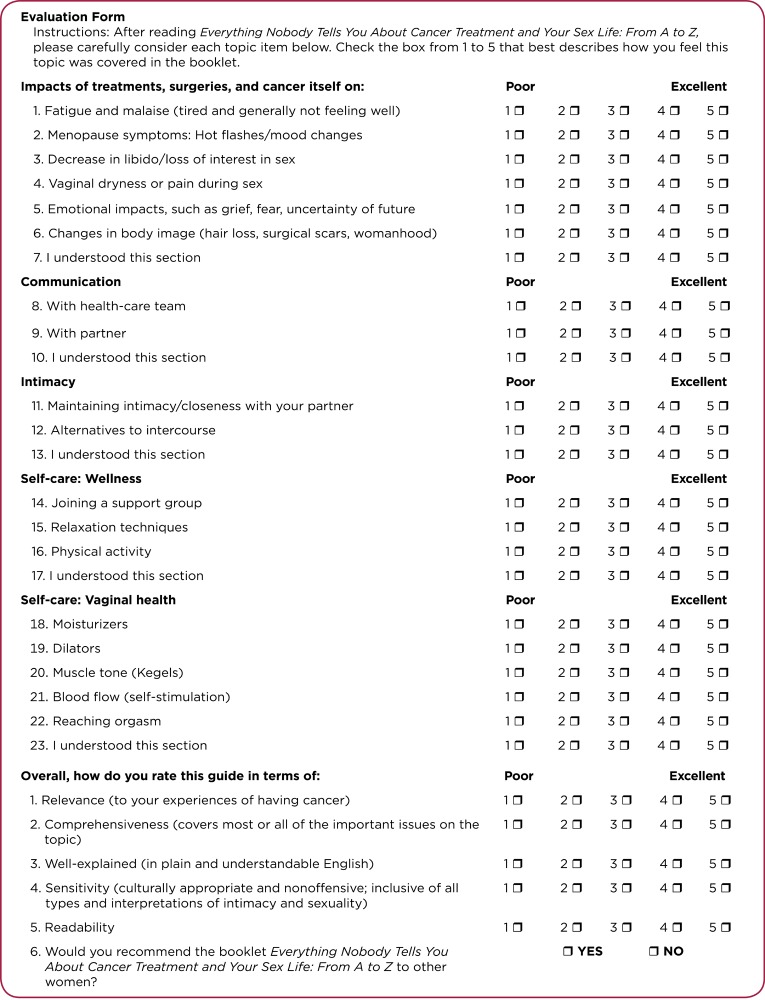
Sample evaluation form for patient resource.

Collaborators who posted the survey on their webpages included Native American Cancer Research; SHOUT; Young Women Cancer Survivors; University of Washington Cancer Center; VAMC Women’s Health Clinic in Seattle, Washington; Anna’s Belles Ovarian Cancer Support Group in Tulsa, Oklahoma; Stephenson Cancer Center at the University of Oklahoma Health Sciences Center; and Camp Mak-A-Dream. Women from any of these groups could refer other women with breast or gynecologic cancer to the webpage to evaluate *The A to Z Guide*. Given this recruitment strategy, we are unable to calculate the response rate.

## RESULTS

The online evaluation was available for 6 months spanning 2011 and 2012, during which 202 responders accessed the survey and 103 women qualified (i.e., had been diagnosed with either breast or gynecologic cancer) to complete the evaluation. Table 1 contains a summary of the demographic characteristics. The average age of the respondents was 51.7 years (range, 29–74; standard deviation [SD] = 10.3), with the average age at diagnosis being 46.2 years (range, 27–67; SD = 9.4). The majority of the sample was Caucasian (97%), married (78%), had a graduate/professional degree (48%), and worked full time (56%). Sixty-nine percent had breast cancer, and 31% had gynecologic cancer. The majority of the respondents had completed treatment with no evidence of disease (73%); 16% of the respondents were newly diagnosed, and 11% had tumor recurrence (9% undergoing treatment and 2% not undergoing treatment).

**Table 1:**
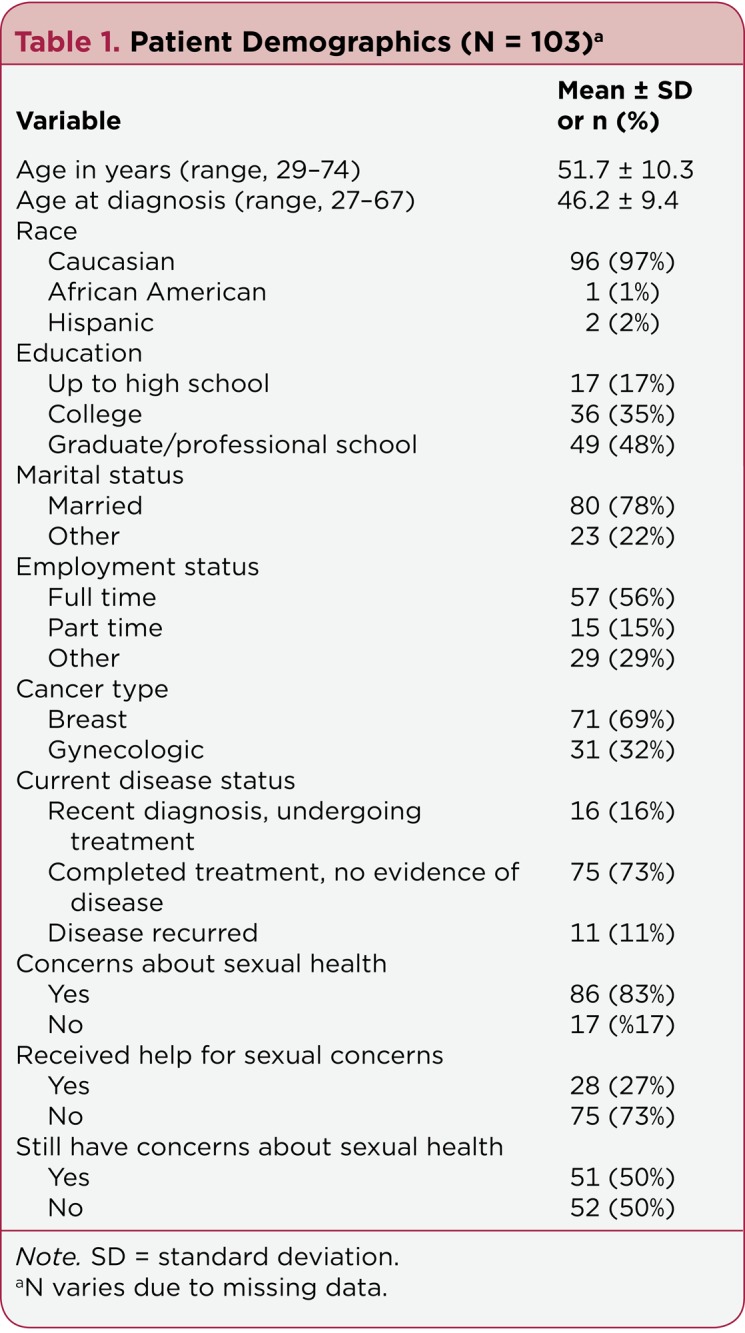
Patient Demographics (N = 103)

The majority of the respondents stated that since their cancer diagnosis, they had had concerns about their sexual health (83%), yet only 27% actually had received help for their sexual health concerns. Those who reported they had received help were asked to describe the help they received. Those who indicated where their help came from said it was from their gynecologist (n = 7), support group (n = 3), therapist (n = 3), a nurse friend (n = 1), a clinical nurse specialist (n = 1), or a social worker (n = 1). And finally, when asked if they still had concerns regarding their sexual health, half of the respondents replied "Yes."

Table 2 contains summary statistics of the respondents’ rating (with 1 = poor and 5 = excellent) for each section. The vaginal health section was considered to be the most understandable (4.59), and the communication section was the least understandable (4.48) yet still very highly rated. The women rated the guide as readable (4.52), understandable (4.50), sensitive (4.30), comprehensive (4.20), and relevant (4.08). Of the respondents, 94% (n = 95) indicated that they would recommend *The A to Z Guide* to other women. The 28-item rating tool for this sample had a high reliability (Cronbach’s alpha = .953).

**Table 2:**
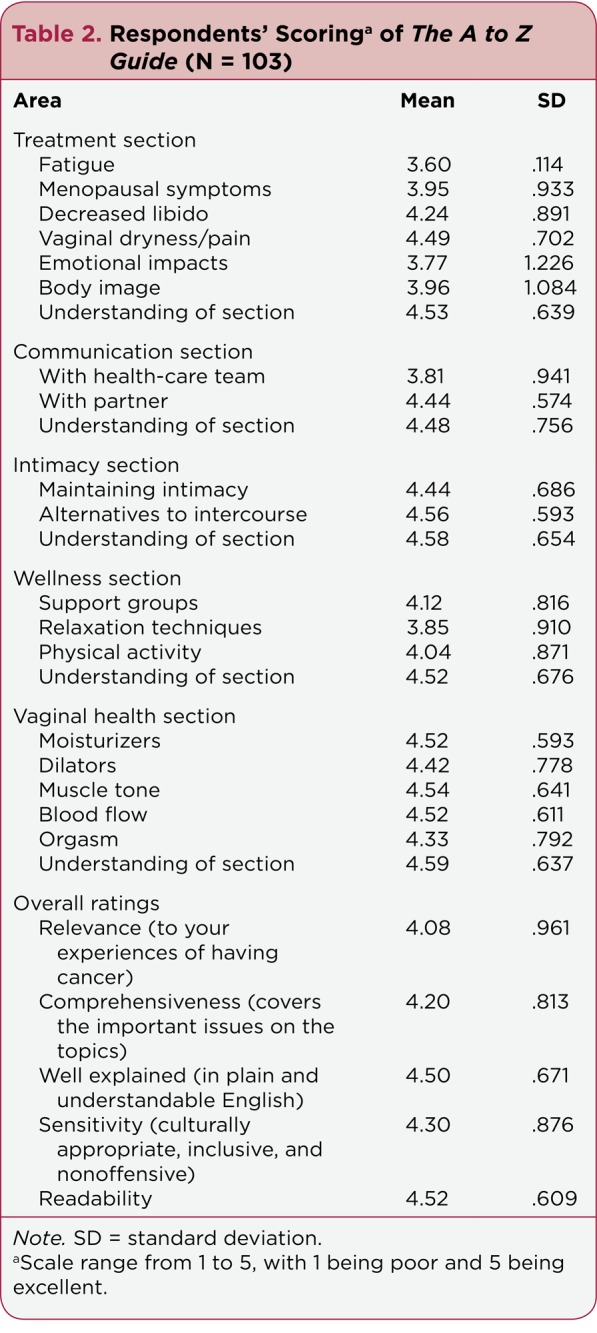
Respondents’ Scoring of The A to Z Guide (N = 103)

We also included open text boxes asking women to advise us if they thought we had left something out of the guide, if something that we included should be removed, and if they had any other suggestions. We compiled 16 single-spaced pages of comments from these text boxes. Many women gave detailed accounts of their experiences regarding their sexual health since diagnoses. Others gave detailed edits to the content, and one woman included a graphic that she recommended be included.

There were 37 comments regarding areas that they thought had been left out, with the predominant topics being decreased libido (n = 4), body image (n = 4), fatigue (n = 3), same-sex relationships (n = 2), and breast stimulation after reconstruction (n = 2). For example, a 46-year-old woman wrote, "You did not really discuss much on the total lack of sex drive. I have found that myself and other ladies with ovarian cancer have a complete and total lack of sex drive. It is difficult at best even to want to try anything mentioned in the book because of this lacking."

One woman commented on body image: "I am having a really hard time with body image. Missing one boob and being hairless and scarred is something I’m worried I can’t get over. I am disgusted every time I look in the mirror."

Another comment focused on same-sex relationships: "You barely mentioned same-sex partners: Are you afraid of the word ’lesbian’? I don’t think you’d be addressing any other variety of same-sex partners. I had the feeling that the entire booklet was aimed at regaining the desire and ability for intercourse and that you mean intercourse when you say lovemaking. How about at least including a paragraph for lesbians?"

One comment on breast stimulation after reconstruction: "Surgery from expanders and a mastectomy with no more nipple stimulation is a big issue for me. Plus, allowing your spouse to look and touch you when you have no feeling in your breasts is a huge adjustment."

Other topics that respondents thought should have been included were changes in orgasm, wearing sexy clothes, manual stimulation, how to talk with their doctors, reproductive health, and the late effects of cancer. For instance, "I think there could have been more discussion, and maybe more explicit information, about manual stimulation by a partner." Another comment was, "I would like to see an addition [or perhaps another book] on issues post-post-post-post cancer diagnosis, effects later on in life. I am 6 years out and wonder whether some of the things I struggle with are normal or not."

Although *The A to Z Guide* team consistently received positive feedback from those who read it, we acknowledge it was written in a style very different from other patient education materials. In part, we conducted the evaluation to see whether the guide would resonate with women living with cancer. There was an open text box that asked women, "Should we remove something from *The A to Z Guide*?" For that question, 17 women wrote, "No." For example, one woman wrote, "No. Everything should be in there. If a person doesn’t want to read it, she can skip it, but others may be interested."

There were four specific comments regarding changes that should be made in future versions. First, there was a comment about using the phrase "final nail in the coffin of your sex life," so we removed that line. Second, there was a reference to something that was said about hugs, and a woman wrote, "Not sure if you should be suggesting to hug everyone and everything, especially in today’s world." Third, there were comments about our primary information relating to heterosexual sex. One woman noted, "I think more attention could be used in addressing same-sex partners and sexual activities. In section F, it is mentioned that your partner can wear a condom and references ’he.’ What about ’she,’ and the use of dental dams? Sexual safety is a big factor for one, and partners may be fearful of fluids/catching cancer as an illness."

The feedback regarding better inclusion of lesbian sexual concerns was valuable, and the investigators therefore sought guidance from colleagues in the LGBT (Lesbian, Gay, Bisexual, and Transgender) community and made major edits throughout the guide (e.g., changing "intercourse" to "sex"). Lastly, in the section about masturbation, a woman said, "On the first page, when you speak to the woman without a partner, you say ’take yourself out to dinner so you don’t feel cheap.’ I would omit this comment, as it may be taken wrong and considered offensive," which we did. This was the only content seen as offensive across all of the evaluations of *The A to Z Guide*. All of the comments were addressed, and revisions were made in the final version.

The final text box offered women the opportunity to offer any other comments or suggestions. The comments included that it was a great resource (n = 16). For example, "I learned a lot. I will refer all the women in my support group to this link. What a great idea this is!" Others discussed what they learned reading *The A to Z Guide* (n = 8). "This is very timely and helpful for me. The comment about how doing nothing is really doing something was a call to action for me." There were also comments related to appreciating the humor in the guide: "Excellent. The humor helped a lot with any anxiety or discomfort. The booklet made the subject seem matter of fact." Another woman wrote, "I enjoyed the humorous style of the booklet. I think that humor can help put people at ease." Other comments related to grammar, sentence structure, the need for art design, and the use of some various time frames.

## DISCUSSION

The evaluation of *The A to Z Guide* and the comments written in the free text boxes were used to guide the final iteration of the document. Through the revision and dissemination of the guide, we have devoted extensive time to ensure that content is presented in clear, understandable direct language without being disrespectful or crude (Mayer, 2014). The women who responded to this evaluation survey overwhelmingly evaluated this educational tool positively.

The women who responded to the survey were predominantly Caucasian, educated, and middle class. Thus, their view may not be representative of minority women living with cancer. Therefore, we cannot generalize our findings to a larger general population. *The A to Z Guide* was written as a resource for women to help them anticipate the sexual health changes their bodies would experience throughout cancer treatment and to validate their experiences throughout survivorship.

Our team has continued to refine *The A to Z Guide* as we receive input from readers. Since the guide was released and made available for download on partner websites, we have received overwhelmingly positive response from users both nationally and internationally. The guide is being used by cancer centers; survivorship clinics; breast, gynecologic, lymphedema, and pelvic radiation support groups; nursing and medical schools; and family medicine clinics. It is our hope that more clinicians will make use of this resource, as it is free and can be downloaded in two formats: one designed for on-screen reading and one collated to print as a booklet.

Oncology professionals are committed to addressing QOL concerns for patients across the trajectory of illness. Sexuality is a key concern for patients, as it impacts relationships and overall QOL (Matzo, 2015). Through careful assessment, patient education, and support, oncology advanced practitioners can ensure that sexuality is respected as an essential part of patient-centered care (Matzo, 2010).
